# Integrated identification of immune-related therapeutic targets for interstitial cystitis via multi-algorithm machine learning: transcriptomic profiling and *in vivo* experimental validation

**DOI:** 10.3389/fimmu.2025.1636855

**Published:** 2025-07-24

**Authors:** Yifan Wang, Chuanzan Zhou, Facai Zhang, Yunkai Yang, Jia Miao, Xuanhan Hu, Xinyu Zhang, Alin Ji, Qi Zhang

**Affiliations:** ^1^ Urology and Nephrology Center, Department of Urology, Zhejiang Provincial People’s Hospital, Hangzhou Medical College, Hangzhou, Zhejiang, China; ^2^ The Second Clinical Medical College, Zhejiang Chinese Medical University, Hangzhou, Zhejiang, China

**Keywords:** interstitial cystitis, machine learning, single-cell analysis, molecular dynamics simulation, *in vivo* experiment

## Abstract

**Background:**

Interstitial cystitis/bladder pain syndrome (IC/BPS) is a complex urological disorder characterized by chronic pelvic pain and urinary dysfunction, with limited diagnostic biomarkers and therapeutic options. Emerging evidence implicates immune microenvironment dysregulation in its pathogenesis, yet the identification of key driver genes and cross-omics integration remains underexplored.

**Methods:**

This study integrated three transcriptomic datasets to identify immune-related gene modules via weighted gene co-expression network analysis (WGCNA). A diagnostic model was constructed using 113 machine learning algorithms. Immune cell infiltration was assessed via CIBERSORT, and single cell sequencing elucidated cellular heterogeneity. Drug candidates were predicted using DSigdb and validated through molecular docking and dynamics simulations. A cyclophosphamide (CYP)/lipopolysaccharide (LPS)-induced IC/BPS murine model was established to evaluate therapeutic efficacy of prioritized compounds (Resiniferatoxin and Acetohexamide) via histopathology, ELISA, and immunohistochemistry.

**Results:**

Eight core immune-related genes were identified. The machine learning model achieved AUC >0.9 in both training and validation cohorts. Single-cell analysis revealed IFI27 overexpression in epithelial and immune cells, correlating positively with M1 macrophages and activated CD4+ T cells (p<0.05). Molecular docking demonstrated strong binding affinity between IFI27 and Acetohexamide (-19.91 ± 0.98 kcal/mol) or Resiniferatoxin (-32.98 ± 1.74 kcal/mol), with dynamics simulations confirming structural stability. *In vivo*, both compounds significantly reduced bladder inflammation (p<0.05), with Acetohexamide showing superior efficacy in downregulating IFI27 expression and systemic pro-inflammatory cytokines.

**Conclusions:**

This multi-omics study deciphered immune dysregulation in IC/BPS and established a robust diagnostic framework. The validation of IFI27-targeting compounds in alleviating inflammation highlights translational potential for repurposed therapeutics. Our findings advance precision immunotherapy strategies for IC/BPS.

## 1 Introduction

Interstitial Cystitis/Bladder Pain Syndrome (IC/BPS) is a complex urological disorder characterized by chronic pelvic pain and urinary frequency/urgency, with a global prevalence ranging from 0.01% to 6.5% and a significantly higher incidence in women than in men (1–3). According to the European Society for the Study of Bladder Pain Syndrome (ESSIC) classification, IC/BPS can be categorized into Hunner-type and non-Hunner-type, with the former accounting for 10%-20% of cases and exhibiting characteristic erythematous mucosal lesions under cystoscopy (4). Although the etiology of IC/BPS remains unclear, studies have identified key pathological features, including disruption of the bladder mucosal barrier, mast cell activation, and neurogenic inflammation (5). Diagnosis primarily relies on clinical symptom exclusion, with the absence of specific biomarkers leading to a misdiagnosis rate of up to 40% and a diagnostic delay of 2-11 years (6). Current treatments, such as oral pentosan polysulfate sodium and intravesical hyaluronic acid instillation, provide only symptomatic relief, while long-term efficacy of immunosuppressants (e.g., cyclosporine) and anti-TNF-α agents is limited, with approximately 10% of patients ultimately requiring bladder augmentation or urinary diversion (7). Therefore, elucidating the molecular mechanisms of IC/BPS and developing precision diagnostic and therapeutic strategies are urgent research priorities.

Recent studies have demonstrated that aberrant immune activation is a critical pathological mechanism in IC/BPS. Single-cell transcriptomic analysis has revealed significant immune cell infiltration in the bladder mucosa of IC/BPS patients, including Th1-polarized CD4+ T cells, reduced regulatory T cells (Tregs), imbalanced M1/M2 macrophage ratios, and abnormal B cell activation (8, 9). Hunner-type patients exhibit a 50-fold increase in plasma cells (CD138+) and a 28-fold increase in B cells (CD20+) in bladder tissue compared to healthy controls, accompanied by elevated urinary IL-6 and TNF-α levels, indicating excessive local humoral immune responses (9). Spatial transcriptomics further reveals that immune cells in IC/BPS are preferentially enriched in the urothelial region, forming an immune-stromal interaction network with fibroblasts through TNF-TNFRSF1B and CD40-TNFSF13B signaling pathways (10). Additionally, autoantibodies against bladder epithelial antigens have been detected in patient sera, suggesting the potential involvement of autoimmune responses in disease progression (11). These findings highlight the highly heterogeneous immune microenvironment of IC/BPS, targeting specific immune subsets or cytokines that emerge as potential therapeutic breakthroughs.

Despite these advances, critical gaps remain in identifying key driver genes and integrating cross-omics data. Traditional methods, such as differential expression analysis, struggle to distinguish disease-associated genes from background noise, whereas machine learning algorithms can enhance the accuracy of feature gene selection through multidimensional data integration (12). In this study, we systematically identified eight core immune-related genes, including IFI27, by integrating transcriptomic data from the GEO database with weighted gene co-expression network analysis (WGCNA) and 113 machine learning algorithms. Then, quantitative real-time PCR (qRT-PCR) experiments validated the expression of key genes. The immune infiltration characteristics of these key genes were analyzed using the CIBERSORT algorithm, and single-cell analysis was employed to explore cellular heterogeneity and the immune microenvironment in IC/BPS. Furthermore, the Drug Signatures Database (DSigdb) predicted acetohexamide and resiniferatoxin as potential therapeutic agents, with molecular docking and molecular dynamics simulations confirming their strong binding affinity to IFI27. Finally, inflammatory factor expression in blood and bladder tissues was measured after administration of the drug to mice, and changes in immunohistochemical sections and IFI27 expression levels in the bladder of mice before and after administration of the drug were detected to determine the efficacy of the drug preliminarily. By combining machine learning with multi-omics data, this study provides novel insights into molecular subtyping of IC/BPS and lays the foundation for targeted drug development and optimization of immunotherapeutic strategies, offering significant clinical translational value.

## 2 Materials and methods

### 2.1 Microarray data acquisition and analysis

We retrieved Interstitial Cystitis/Bladder Pain Syndrome (IC/BPS)-related datasets from the Gene Expression Omnibus (GEO) database and downloaded three independent public datasets (GSE11783, GSE28242, and GSE57560). To address batch effects and ensure data consistency, we first performed batch effect correction using the “SVA” R package. The datasets from each group were merged, and principal component analysis (PCA) was applied to reduce dimensionality and eliminate potential batch effects. The integrated dataset includes 31 IC/BPS samples (10 from GSE11783, 8 from GSE28242, and 13 from GSE57560) and 14 normal bladder samples (6 from GSE11783, 5 from GSE28242, and 3 from GSE57560). Subsequently, differential expression analysis was conducted on the integrated dataset using the “Limma” R package, identifying differentially expressed genes (DEGs) that met the criteria of |logFC| > 1 and p.adjust < 0.05. These DEGs were visualized as a heatmap using the “Pheatmap” package.

Immune-related genes were obtained from the GeneCards database (https://www.genecards.org/) using the screening criteria of “Protein Coding” and a relevance score > 2, yielding 8,314 related genes. A Venn diagram was employed to identify the intersection between immune-related genes and IC/BPS-specific DEGs, thereby pinpointing immune-related DEGs associated with IC/BPS.

### 2.2 Construction of weighted gene co-expression networks and identification of key module genes

WGCNA constructs scale-free networks by correlating gene expression levels with clinical traits. Using the “WGCNA” R package, we first removed apparent outliers from the dataset. An optimal soft threshold was selected to generate an adjacency matrix based on the topological overlap matrix (TOM). Subsequently, genes with similar expression patterns were clustered into gene modules through average linkage hierarchical clustering, using TOM-based dissimilarity measures. Finally, the modules with the most significant positive and negative correlations to clinical traits were identified. Module membership (MM) and gene significance (GS) were calculated to assess the relationships between co-expressed genes and clinical traits. Genes with higher MM and GS values exhibited stronger correlations with their respective modules and clinical traits. Applying the screening criteria of MM > 0.5 and GS > 0.5, we identified key genes within the most relevant modules.

### 2.3 Functional enrichment analysis and gene set variation analysis

To gain deeper insights into the functional roles of immune-related IC/BPS-DEGs, Gene Ontology (GO) and Kyoto Encyclopedia of Genes and Genomes (KEGG) enrichment analyses were performed using the “ClusterProfiler” R package. GO enrichment analysis, a widely used bioinformatics tool categorizes gene functional annotations into molecular functions, biological processes, and cellular components (CC). Similarly, KEGG pathway enrichment analysis has been extensively employed to elucidate biological mechanisms and functions (13). A threshold of p < 0.05 was considered statistically significant for enrichment. The results were visualized using circle plots and the “GOplot” package.

The biological significance of IC core genes was evaluated using the “GSVA” R package based on gene sets from the MSigdb. This comprehensive approach provided a detailed understanding of the functional pathways and biological processes associated with immune-related IC/BPS-DEGs, offering valuable insights into the underlying mechanisms of IC/BPS.

### 2.4 Integrative machine learning algorithms constructed an optimal model

Based on the intersection feature genes identified through WGCNA, we employed an integrated machine learning approach to screen for core genes associated with IC/BPS. A total of 11 classical algorithms were utilized, including Lasso, Stepglm, glmBoost, SVM, Ridge, Enet, plsRglm, Random Forest, LDA, XGBoost, and NaiveBayes. The process for generating immune-related IC/BPS signatures (IRICs) was as follows: Within the test set, prediction models were fitted using a leave-one-out cross-validation framework with 113 algorithm combinations; All models were validated across three GEO cohorts (3); For each model, the Harrell’s concordance index (C-index) was calculated across all test sets and GEO datasets, and the model with the highest average C-index was selected as the optimal one. Additionally, receiver operating characteristic (ROC) curves were constructed for each diagnostic feature in the training and validation sets. The area under the curve (AUC) values were computed to evaluate the diagnostic performance of IRICs in distinguishing normal samples. To further quantify model performance, a confusion matrix was introduced, comparing predicted values with actual values to optimize the gene selection process. This rigorous approach ensured that the selected genes were both biologically meaningful and statistically reliable.

### 2.5 Immune cell infiltration assessment and correlation analysis

To investigate the immunological characteristics between IC/BPS samples and controls, we employed the CIBERSORT algorithm to quantify the infiltration levels of 28 immune cell types and to assess the correlations among these cells. Furthermore, Spearman’s rank correlation test was utilized to evaluate the relationships between each core gene and immune cell types in IC/BPS samples. This comprehensive analysis provided insights into the immune microenvironment and its potential role in IC/BPS pathogenesis.

### 2.6 Single-cell RNA-seq data collection and processing

In this study, single-cell RNA sequencing (scRNA-seq) data from the GSE175526 dataset were processed using the “Seurat” R package. Cells were filtered based on gene counts and the percentage of mitochondrial genes, excluding those with too few genes or excessively high mitochondrial gene content. The “NormalizeData” function was employed for data normalization, and the “FindVariableFeatures” function was used to identify the top 1,500 highly variable genes. Principal component analysis (PCA) was performed on the normalized data using the “RunPCA” function. Batch effects across different samples were corrected using the “Harmony” function, followed by dimensionality reduction and cluster identification via uniform manifold approximation and projection (UMAP). The “SingleR” package was utilized to annotate different cell clusters into specific cell subpopulations. Subsequently, the expression levels of core biomarkers across various cell groups were analyzed and visualized. Finally, cell-cell interaction analysis was conducted using the “CellChat” package, providing insights into the intercellular communication networks within the IC/BPS microenvironment.

### 2.7 Verification of key gene expression

Total RNA was extracted from mouse bladder tissues using the SPARKeasyImprovedTissue/Cell RNA Extraction Kit II (AC0202, Shandong Sparkjade Biotechnology Co., Ltd.). Reverse transcription of mRNA was performed using the Evo M-MLV Plus 1st Strand cDNA Synthesis Kit (AG11615, ACCURATE BIOTECHNOLOGY (HUNAN) CO., LTD, Changsha, China). qRT-PCR was conducted using the GeniuScript III One Step Greener RT-qPCR Kit (Q1009A, U&G Biotech). Relative expression levels were calculated using the 2-ΔΔCT method. Specifically designed primers are listed in **Supplementary Table 1**.

### 2.8 Potential drug prediction and molecular docking identification

To further explore the clinical significance of IRICs, we utilized the DSigdb to predict small-molecule drugs with potential therapeutic effects. Protein crystal structures for docking were obtained from the AlphaFold database, while the 3D structures of small molecules were downloaded from the PubChem database and energy-minimized under the MMFF94 force field. Molecular docking was performed using AutoDock Vina 1.2.3. Prior to docking, all receptor proteins were processed with PyMol 2.5.5, including the removal of water molecules, salt ions, and small molecules. The docking box was then defined using the PyMol plugin center_of_mass.py, with the box center based on the active site location and the box edge length set to 22.5 Å. Additionally, ADFRsuite 1.0 was employed to convert the processed small molecules and receptor proteins into the PDBQT format required for AutoDock Vina 1.2.5. During docking, the exhaustiveness of the global search was set to 32, while other parameters remained at their default settings. The highest-scoring docking conformation was selected as the binding conformation for subsequent molecular dynamics simulations.

### 2.9 Validation of molecular dynamics simulations

Based on the small molecule-protein complexes obtained from the aforementioned docking results, all-atom molecular dynamics (MD) simulations were performed using the AMBER 22 software. Prior to the simulation, the charges of the small molecules were calculated using the antechamber module and Gaussian 09 software with the Hartree–Fock (HF) SCF/6-31G* method. The small molecules and proteins were described using the GAFF2 force field and the ff14SB protein force field, respectively. Hydrogen atoms were added to the systems using the LEaP module, and a truncated octahedral TIP3P water box with a 10 Å cutoff was added to solvate the systems. Na+/Cl- ions were incorporated to neutralize the system charge, and the topology and parameter files for the simulation were generated.

The MD simulations were conducted using AMBER 22. The systems were first energy-minimized using 2,500 steps of steepest descent followed by 2,500 steps of conjugate gradient minimization. Subsequently, the systems gradually heated from 0 K to 298.15 K over 200 ps under constant volume and controlled temperature. After reaching the target temperature, the systems were equilibrated for 500 ps under the NVT (canonical) ensemble to ensure uniform solvent distribution. Finally, the systems were equilibrated for 500 ps under the NPT (isothermal-isobaric) ensemble. The production run was performed for 100 ns under the NPT ensemble with periodic boundary conditions. During the simulation, a non-bonded cutoff of 10 Å was applied, long-range electrostatic interactions were calculated using the Particle Mesh Ewald (PME) method, hydrogen bond lengths were constrained using the SHAKE algorithm, and temperature control was achieved using the Langevin thermostat with a collision frequency (γ) of 2 ps⁻¹. The system pressure was maintained at 1 atm, and the integration time step was set to 2 fs. Trajectories were saved every 10 ps for subsequent analysis.

The binding free energies between the proteins and ligands in all systems were calculated using the MM/GBSA method. To ensure accuracy in MM/GBSA calculations, MD trajectories from 90 to 100 ns were used for the analysis. The specific formula for the calculation is as follows:


(1) $$\begin{array}{l} \text{Δ}G_{bind}={\text{ΔG}}_{\text{complex}}\;-\;\left({\text{ΔG}}_{\text{receptor}}+{\text{ ΔG}}_{\text{ligand}}\right)\\ \;={\text{ΔE}}_{\text{internal}}+{\text{ΔE}}_{\text{VDW}}+{\text{ΔE}}_{\text{elec}}+{\text{ΔG}}_{\text{GB}}+{\text{ΔG}}_{\text{SA}} \end{array}$$

In Equation 1, ΔE_internal_ represents the internal energy, ΔE_VDW_ denotes the van der Waals interactions, and ΔE_elec_ corresponds to the electrostatic interactions. The internal energy includes contributions from bond energy (E_bond_), angle energy (E_angle_), and torsional energy (E_torsion_). ΔG_GB and ΔG_SA collectively represent the solvation free energy, where ΔG_GB_ is the polar solvation free energy and ΔG_SA_ is the nonpolar solvation free energy. For ΔG_GB_, the GB model developed by Nguyen et al. (igb = 2) was employed. The nonpolar solvation free energy (ΔG_SA_) was calculated based on the product of the surface tension (γ) and the solvent-accessible surface area (SASA), as ΔG_SA_ = 0.0072 × ΔSASA. Due to the high computational cost and low accuracy associated with entropy calculations, entropy changes were not considered in this study.

### 2.10 Animal models

An interstitial cystitis/bladder pain syndrome (IC/BPS) model was established in 24 female C57BL/6 mice (10-week-old) through cyclic intravesical instillation of cyclophosphamide (CYP, 35 mg/kg) and lipopolysaccharide (LPS, 0.6 mg/kg) via transurethral catheterization under 1.5% isoflurane anesthesia. Animals were randomized into four groups (n=6/group): IC/BPS model, saline-instilled control, resiniferatoxin-treated (60 μg/kg), and acetohexamide-treated (30 mg/kg) (14). Following urethral catheterization using a 24G indwelling needle with stylet guidance, chemical instillation was performed every 3 days for five cycles, with saline flushing 30 min post-administration. Two weeks post-modeling, the same regimen was used for intravesical perfusion of the therapeutic agent. Urine samples were collected during 48-h metabolic cage housing post-treatment prior to CO_2_ euthanasia. Bladder tissues and blood were harvested for pathological evaluation, with inflammatory cytokines (IL-6, TNF-α) quantified using QuantiCyto^®^ ELISA kits (Neobioscience: EMC004(H).96.2, EMC102a.96.2). Fixed paraffin-embedded bladder sections underwent hematoxylin-eosin and immunohistochemical staining, with microscopic imaging conducted using an Olympus BX53 system. All procedures complied with institutional animal ethics guidelines (Hospital Animal Ethics Committee Approval No. 20250304829577).

### 2.11 Statistical analysis

All statistical analyses were performed using Perl version 5.32.1 and R software version 4.4.1. Additionally, GraphPad Prism 8.0 (GraphPad Software Inc., USA) was employed for statistical evaluation and the creation of visual data displays. The ELISA results were analyzed by one-way ANOVA. A P-value of <0.05 was considered statistically significant for all analyses.

## 3 Results

### 3.1 Microarray data collection and preprocessing

The three microarray datasets, GSE11783, GSE28242, and GSE57560, were normalized and merged into a large training/internal validation cohort. Batch effects were corrected using the “RemoveBatchEffect” function from the “Limma” package (**Supplementary Figures 1A, B**). After normalization, the data distribution of each dataset fell within a similar range, and batch effects between datasets were effectively mitigated (**Supplementary Figures 1C, D**).

### 3.2 Identification of DEGs in IC/BPS

DEGs were identified between 31 IC/BPS cases and 14 normal controls using the “limma” R package. To explore immune-related genes, we integrated genes obtained from the GeneCards database and relevant literature with the identified DEGs. As a result, 73 immune-related differentially expressed genes were identified. These findings were visually represented in a heatmap (**Supplementary Figure 2**).

### 3.3 Evaluating key modules in weighted gene co-expression network

**Figure 1:**
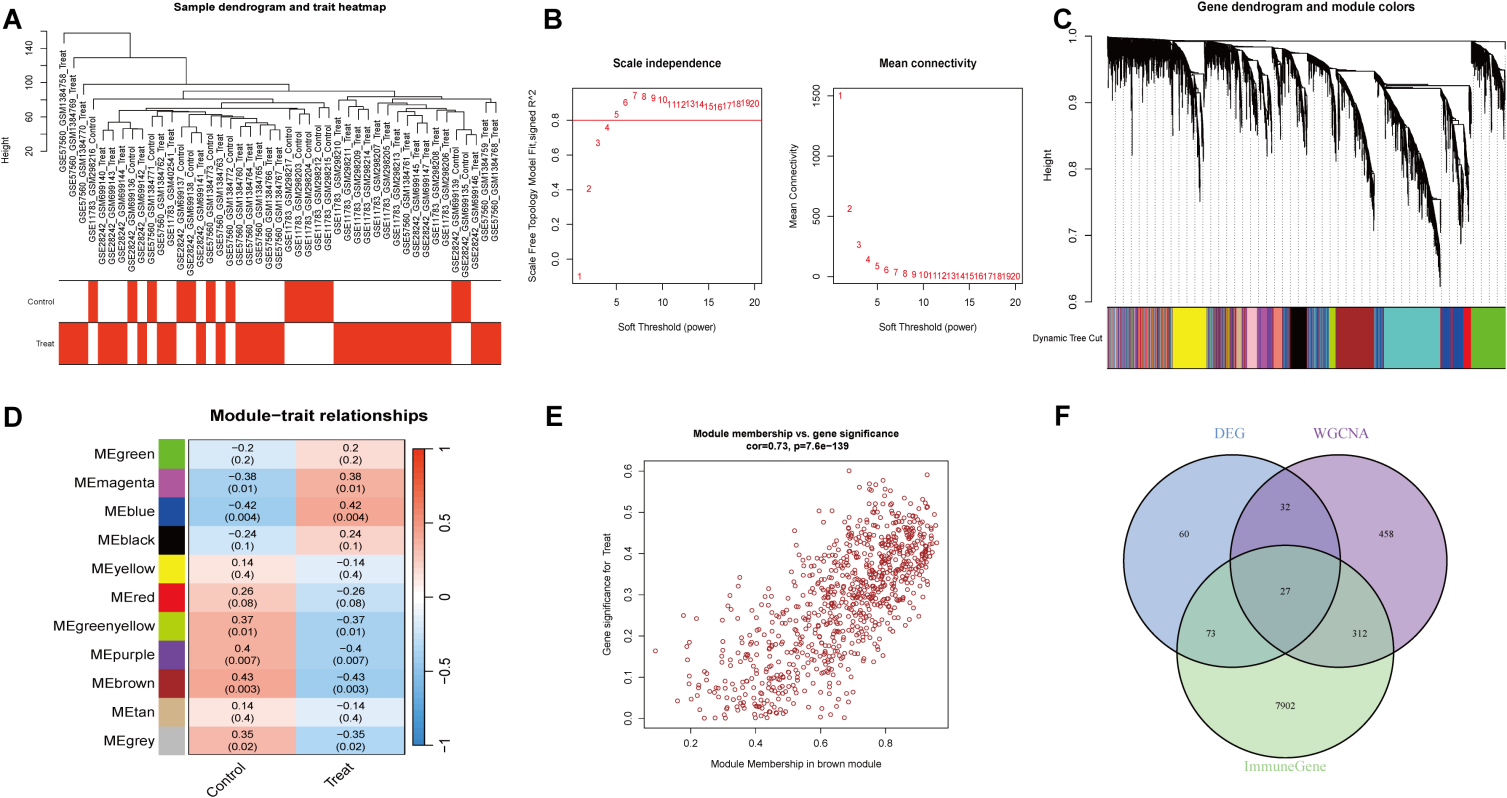
WGCNA screening for immune-related genes. **(A)** Sample clustering tree. **(B)** Soft threshold power and average connectivity of WGCNA. **(C)** Clustering tree diagram. **(D)** Heatmap depicting the relationship between modules and clinical features, especially IC/BPS and control. **(E)** Scatter plot depicting the relationship between module affiliation (MM) and gene significance (GS) in brown modules. **(F)** Venn diagram of the intersection of DEGs, brown component genes, and immune-related candidate genes.

To comprehensively identify key genes associated with IC/BPS phenotypes, we performed WGCNA. Hierarchical clustering of all samples was conducted, and outliers were excluded, as shown in the sample dendrogram (**Figure 1A**). A soft threshold power (β) of 6 was selected to construct a scale-free topological network (**Figure 1B**). In this study, the minimum module gene count was set to 60, and the MEDissThres was set to 0.25, ultimately identifying 11 co-expression modules (**Figure 1C**). Our results revealed that the MEbrown module exhibited the strongest positive correlation with IC/BPS in the cohort (cor = 0.43, p = 0.003, **Figure 1D**). Additionally, the gene significance (GS) of the MEbrown module was significantly correlated with module membership (MM) (cor = 0.73, p < 7.6e-139, **Figure 1E**). These findings suggest that genes within the MEbrown module may have functional relevance to IC/BPS. Based on the criteria of GS > 0.5 and MM > 0.5, we screened 829 key genes from the MEbrown module. By intersecting these with previously identified DEGs, we filtered out 27 Immune-related IC/BPS-DEGs (**Figure 1F**).

### 3.4 Functional enrichment analysis

To elucidate the immune response mechanisms in IC/BPS, functional enrichment analysis was performed on the initially screened genes (**Supplementary Figure 3**). GO analysis revealed that immune-related IC/BPS-DEGs were significantly enriched in biological processes such as defense response to viruses, regulation of phagocytosis, and humoral immune response, and played important roles in immunoglobulin binding and cytokine receptor activity. Additionally, KEGG pathway analysis identified significant associations with ABC transporters and antifolate resistance. These enrichment results suggest the presence of a high level of stress and abnormal immune response activation during the progression of IC/BPS.

### 3.5 Construction and validation of the diagnostic signatures based on integrative machine learning

**Figure 2:**
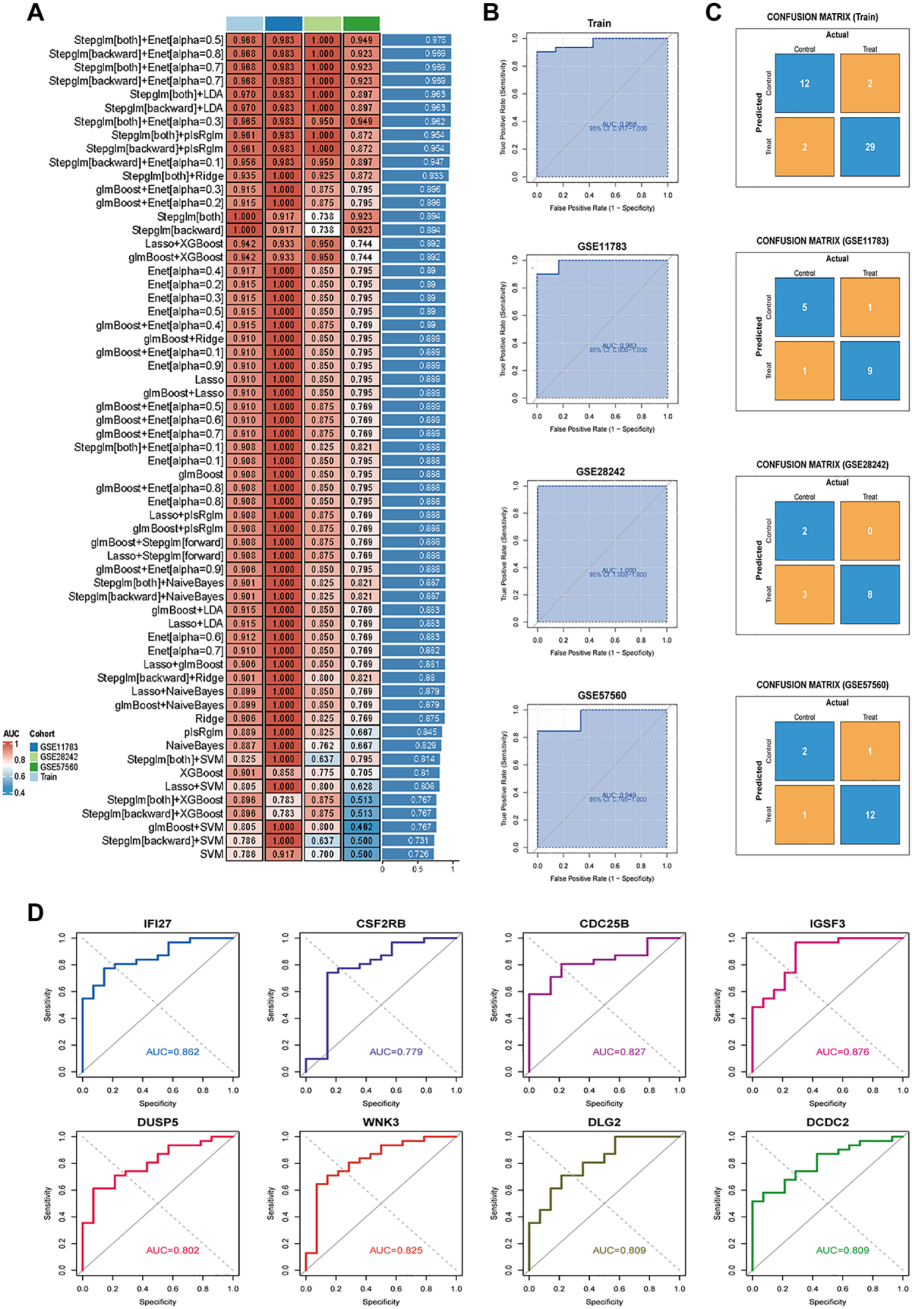
Constructing and validating diagnostic features through integrated machine learning. **(A)** 113 combinations of predictive models using 10-fold cross-validation and graded AUC indices. **(B)** Coefficients of Stepglm[both]+Enet[alpha=0.5] visualization and diagnostic features. **(C)** Confusion matrix plots for the training and validation sets. **(D)** ROC plots for each diagnostic feature.

To construct an IRIC-based diagnostic signature, we employed 113 combinations of 11 machine learning algorithms for variable selection and model development. The integrated cohort was divided into a training set and an internal validation set in a 7:3 ratio to ensure balanced distribution of clinical features. Within the internal training set, 10-fold cross-validation was performed to evaluate each algorithm combination and calculate AUC values. The ranking of AUC values for all algorithms is shown in **Figure 2A**. Notably, the Stepglm[both]+Enet[alpha=0.5] combination demonstrated optimal performance across both internal and external datasets, with AUC values of 0.968 (95% CI: 0.912–1.000), 0.983 (95% CI: 0.900–1.000), 1.000 (95% CI: 1.000–1.000), and 0.949 (95% CI: 0.795–1.000) (**Figure 2B**). The confusion matrix can also be seen in **Figure 2C**, where the overall accuracy of the model on the training set is as high as (12 + 29)/(12 + 2 + 2+29) = 91.1%. The model shows strong classification ability on all the sets with high overall prediction accuracy. Eight core genes were identified: IFI27, CSF2RB, CDC25B, IGSF3, DUSP5, WNK3, DLG, and DCDC2. The AUC of IRICs surpassed that of individual gene diagnostic features, suggesting its potential benefit for IC/BPS patients (**Figure 2D**).

### 3.6 Validation of core gene expression in IC/BPS

**Figure 3:**
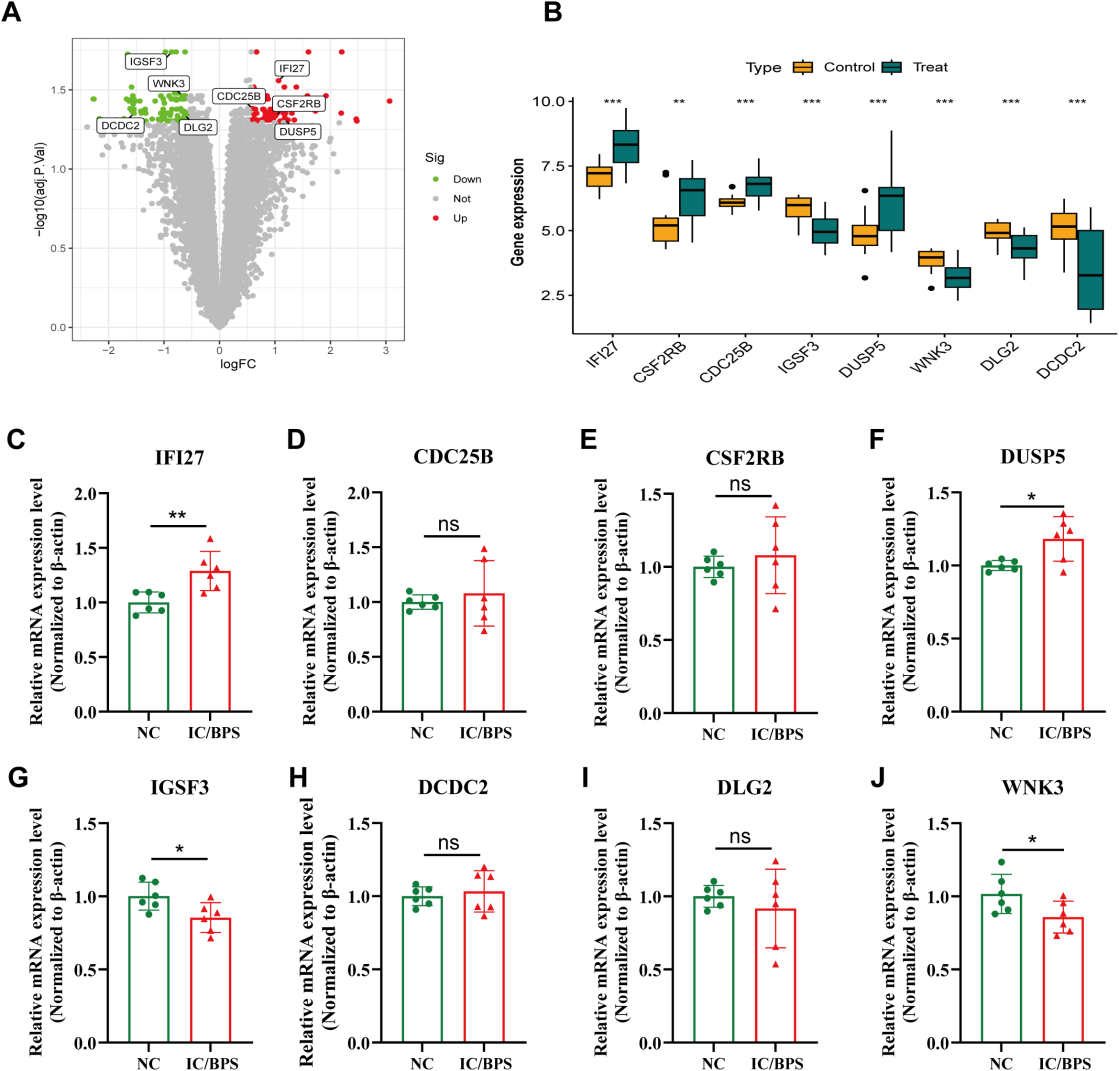
Identification of 8 candidate hub genes. **(A)** Volcano plot of IRICs between control and IC/BPS samples. **(B)** Expression analysis of IRICs in IC/BPS and controls based on the GEO dataset. **(C–J)** The mRNA expression levels of IRICs were verified using qRT-PCR. *p < 0.05; **p < 0.01; ***p < 0.001; ns, no statistical difference.

The volcano plot further revealed the differential expression of the 8 core genes, including 4 up-regulated and 4 down-regulated genes. The boxplot demonstrated that IFI27, CSF2RB, CDC25B, and DUSP5 were significantly up-regulated, while IGSF3, WNK3, DLG2, and DCDC2 were down-regulated (**Figures 3A, B**). The mRNA expression levels of the 8 core genes were further validated using qRT-PCR. Compared to the control group, the expression levels of IFI27 and DUSP5 were significantly up-regulated in the IC/BPS group (**Figures 3C, F**). Conversely, the expression of IGSF3 and WNK3 was significantly down-regulated in the IC/BPS group (**Figures 3G, J**). The mRNA expression of the remaining genes showed some differences but did not reach statistical significance (**Figures 3D, E, H, I**). These results indicate that the expression levels of the 8 core genes are largely consistent with the bioinformatics analysis.

### 3.7 GSVA enrichment analysis of IRICs

**Figure 4:**
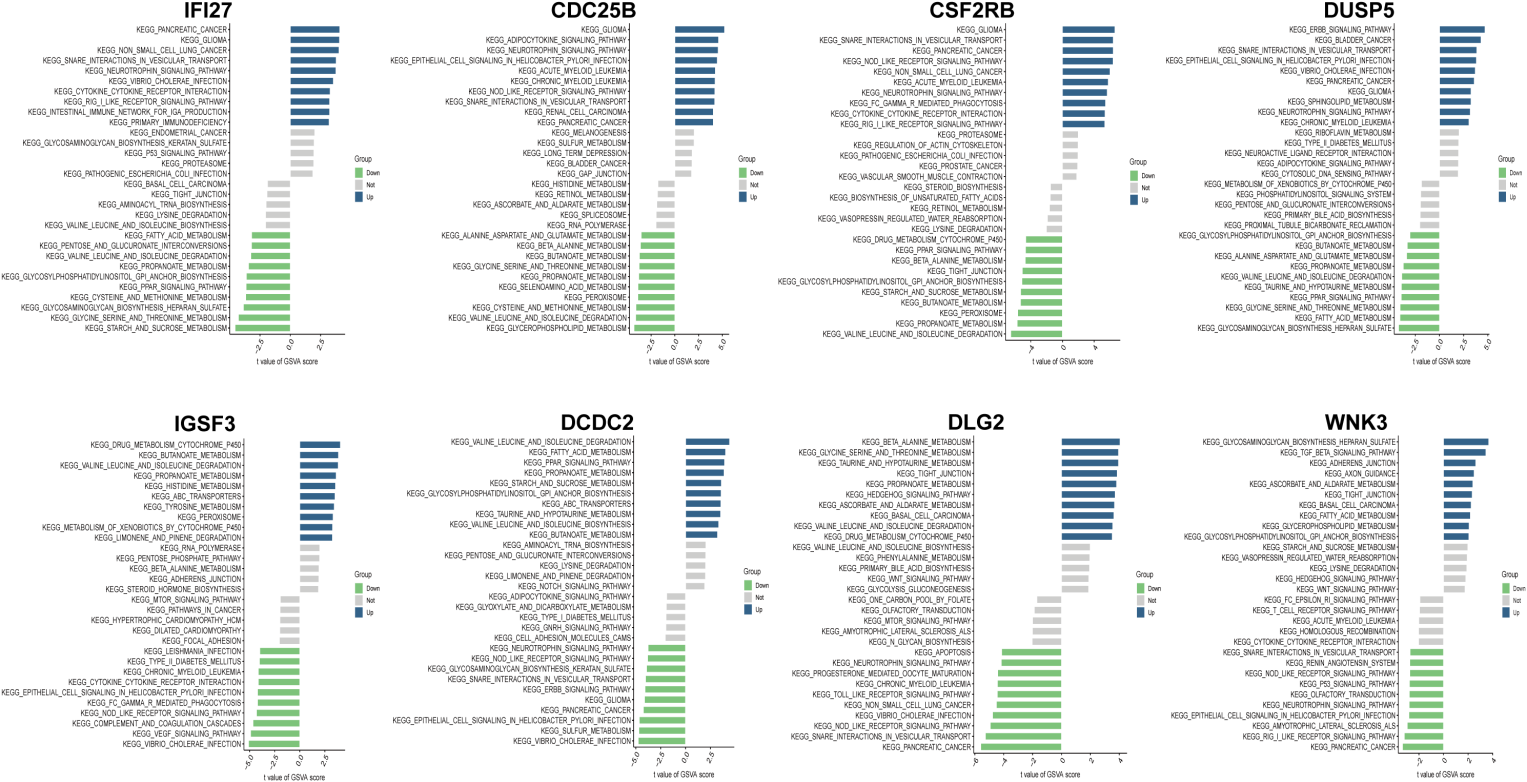
GSVA enrichment analysis of IRICs.

To elucidate the potential signaling pathways and mechanisms underlying IC/BPS, pathway enrichment analysis was performed on both up- and down-regulated IRICs. GSVA of IRICs indicated that upregulated pathways were primarily involved in cytokine-cytokine receptor interaction, RIG-I-like receptor signaling, and NOD-like receptor signaling, implying their potential roles in metabolic-immune crosstalk or stress responses (**Figure 4**).

### 3.8 Immune infiltration analysis and association with diagnostic signatures

**Figure 5:**
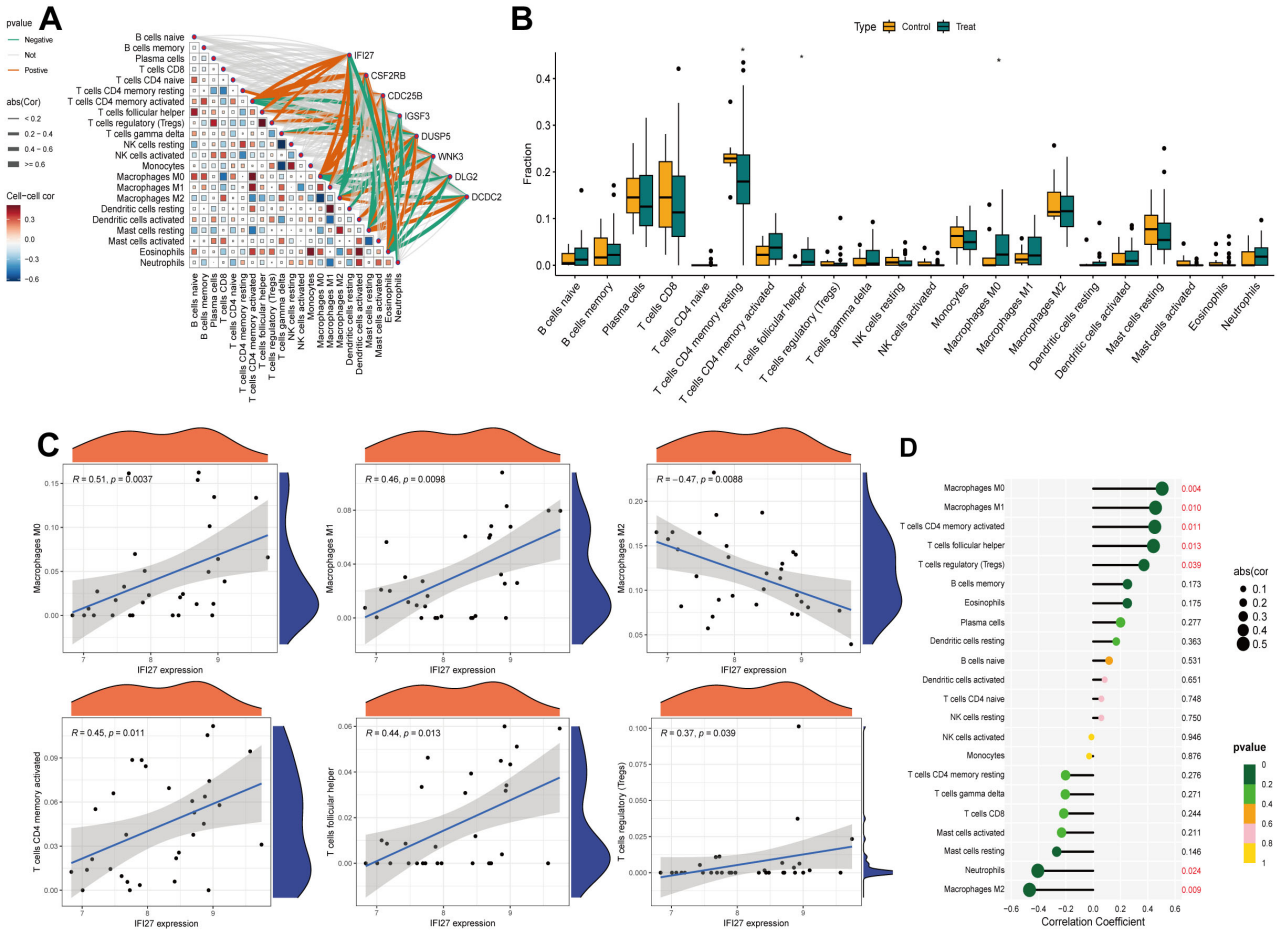
Immune infiltration analysis of IRICs. **(A)** 22 differences in immune infiltration of immune cells between the two groups. **(B)** ET-Link plots of different hub genes correlating with immune cells. **(C, D)** Analysis of the correlation between IFI27 and immune cells. *p < 0.05.

We employed the CIBERSORT algorithm to assess the correlation between the expression profiles of 22 immune cell types in IC/BPS and normal groups. Based on immune infiltration analysis and intergroup comparison boxplots, we analyzed and visualized the infiltration abundance of 22 immune cell types in both groups (**Figure 5A**). Significant differences were observed in the expression of T cells CD4 memory activated, T cells follicular helper, and Macrophages M0 between the two groups. Subsequently, the correlation between each core gene and immune cells was displayed in a correlation network heatmap (**Figure 5B**). Notably, the IFI27 gene showed significant positive correlations with Macrophages M0, Macrophages M1, T cells CD4 memory activated, T cells follicular helper, and T cells regulatory (Tregs), while exhibiting a negative correlation with Macrophages M2 (**Figures 5C, D**).

### 3.9 Single cell analysis for IRICs in IC/BPS

**Figure 6:**
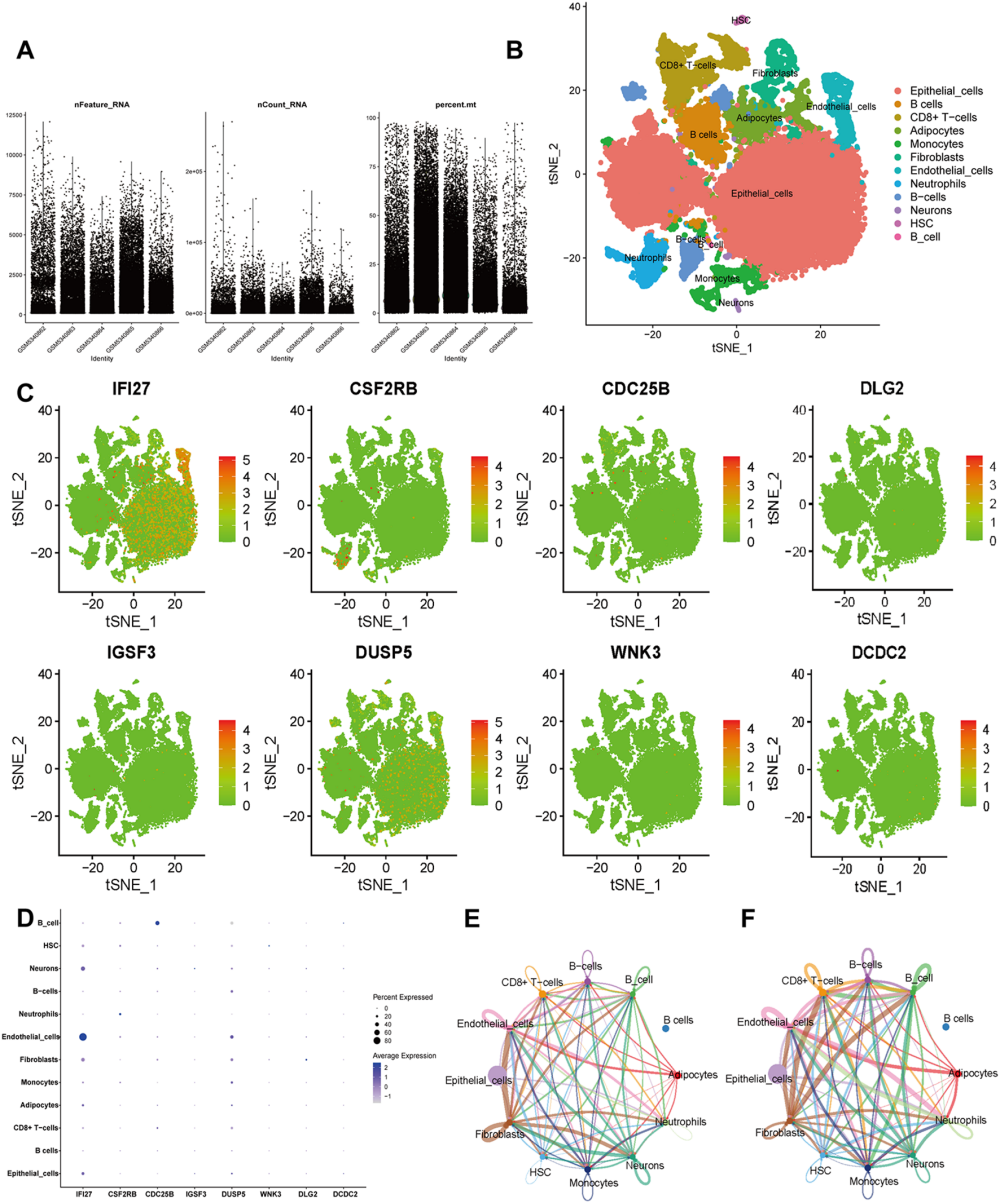
Single-cell RNA sequencing (scRNA-seq) in GSE175526. **(A)** Scatter plots of characteristic genes regarding sample gene number, sequencing depth, and mitochondrial content. **(B)** Cluster analysis identified 10 distinct cell clusters, which were visualized and annotated using t-distribution random neighbor embedding (t-SNE). **(C)** The t-SNE plot illustrates the expression trends of biomarkers across cell types. **(D)** Bubble plot displaying the expression patterns of biomarkers across cell types. **(E, F)** Cellular communication networks illustrate the number **(E)** and strength **(F)** of interactions.

To comprehensively characterize the feature genes, we performed single-cell analysis using the GSE175526 dataset (**Figure 6A**). The data were clustered using the “Seurat” R package. Visualization via UMAP identified 10 distinct cell types, including endothelial cells, epithelial cells, fibroblasts, monocytes, neuronal cells, hematopoietic stem cells, adipocytes, neutrophils, B cells, and CD8+ T cells (**Figure 6B**). The expression patterns of each core gene in different cell types were then investigated, and t-SNE visualization showed that IFI27 exhibited dense expression in most cell types (**Figure 6C**). Dot plot analysis further demonstrated that IFI27 was highly expressed in epithelial cells, endothelial cells, and fibroblasts, with partial expression in monocytes and CD8+ T cells (**Figure 6D**). To explore biological feature differences, we investigated complex communication networks among the 10 annotated cell types. The results indicated increased communication strength between CD8+ T cells (as ligand cells) and monocytes as well as neutrophils. Monocytes also exhibited strong communication with endothelial cells, neutrophils, and B cells (**Figure 6E**). Additionally, the number of interactions between fibroblasts and other cell types was notably increased (**Figure 6F**).

### 3.10 Prediction of potential small molecule drugs

**Figure 7:**
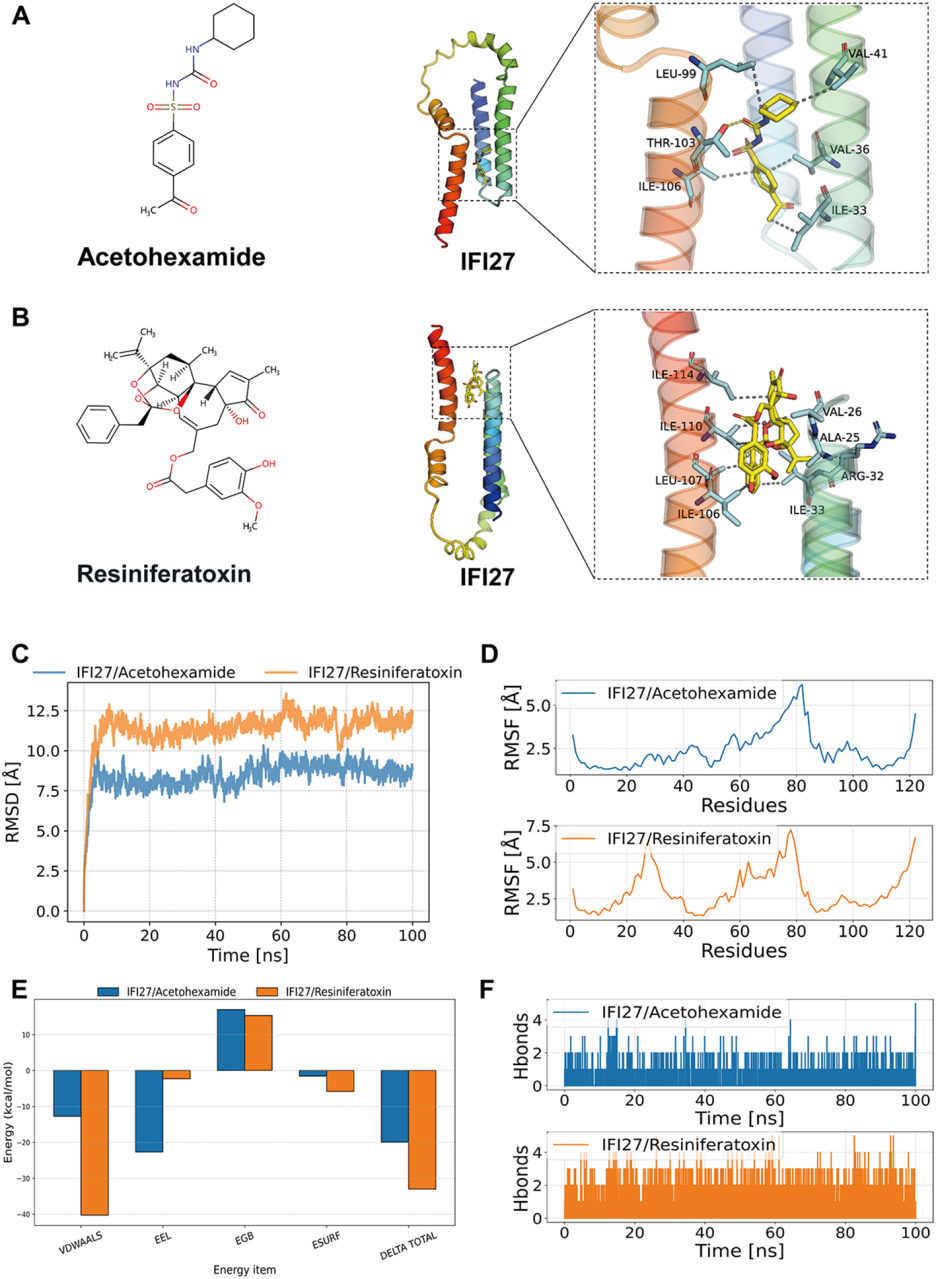
Molecular docking and molecular dynamics simulation results. **(A, B)** Binding patterns of IFI27 with Acetohexamide and Resiniferatoxin were obtained based on docking. The left figure shows the overall view and the right figure shows the partial view, in which the yellow stick is the small molecule, the cyan cartoon is the protein, the blue line indicates the hydrogen bonding interaction, and the magenta dashed line indicates the salt bridge interaction. **(C)** RMSD of the complex over time during molecular dynamics simulations. **(D)** RMSF is calculated based on molecular dynamics simulation trajectories. **(E)** MM-GBSA binding energy and energy decomposition. **(F)** Change in the number of hydrogen bonds between small molecules and proteins during molecular dynamics simulations.

Molecular docking simulation is a convenient and effective approach for exploring the interactions between small molecules and target proteins. Here, we used Vina 1.2.3 software to perform docking studies on the compounds Acetohexamide, Resiniferatoxin, and the IFI27 protein. As shown in **Figure 7A**, hydrogen bond interactions were observed between the small molecules and THR-103 on the protein, while hydrophobic interactions occurred with ILE-106, LEU-99, VAL-41, ILE-33, and VAL-36. The formation of hydrogen bonds and hydrophobic interactions enhanced the binding affinity between the protein and small molecules. The interactions of the IFI27_Acetohexamide complex are illustrated in **Figure 7B**, revealing hydrogen bond formation with THR-103 and hydrophobic interactions with ILE-106, LEU-99, VAL-41, MET-44, and VAL-18 on the protein.

### 3.11 Molecular dynamics modeling of potential drugs and targets

The root mean square deviation (RMSD) in molecular dynamics simulations reflects the motion of complexes, where higher RMSD values and more pronounced fluctuations indicate greater motion, and vice versa. As shown in **Figure 7C**, the RMSD changes of the IFI27/Acetohexamide and IFI27/Resiniferatoxin complexes during the simulation are presented. During the simulation, the RMSD of the IFI27/Acetohexamide complex increased rapidly in the 0–20 ns range and then stabilized, maintaining fluctuations within 7–10 Å throughout the simulation. In contrast, the RMSD of the IFI27/Resiniferatoxin complex started from a lower initial value, continued to rise within the first 40 ns, and eventually stabilized at approximately 8 Å. The two complexes exhibited different RMSD trends during the dynamics process, indicating differences in their dynamic stability. Although both complexes showed relatively stable RMSD values in the later stages, the IFI27/Resiniferatoxin complex exhibited significantly smaller fluctuations, suggesting higher structural stability.

The root mean square fluctuation (RMSF) reflects the flexibility of proteins during molecular dynamics simulations. Typically, drug binding reduces protein flexibility, thereby stabilizing the protein and enhancing enzymatic activity. As shown in **Figure 7D**, the RMSF values of all proteins bound to different small molecules were low (<5 Å), except at the terminal regions, indicating good rigidity of the protein core structure. In summary, the low flexibility of the protein is the foundation for stable binding between small molecules and the protein.

Based on the molecular dynamics simulation trajectories, we calculated the binding energy using the MM-GBSA method, which more accurately reflects the binding modes of small molecules to the target protein (**Figure 7E**). As shown in **Table 1**, the binding energies of the IFI27/Acetohexamide and IFI27/Resiniferatoxin complexes were −19.91 ± 0.98 and -32.98 ± 1.74 kcal/mol, respectively. Negative values indicate binding affinity between the molecules and the target protein, with lower values indicating stronger binding. Our calculations clearly demonstrate that these molecules have a binding affinity with the corresponding proteins, with IFI27/Resiniferatoxin exhibiting higher binding energy. The binding energy of these complexes is primarily contributed by van der Waals and electrostatic interactions, while solvation energy is unfavorable for their binding.

**Table 1:** Binding free energies and energy components predicted by MM/GBSA (kcal/mol).

System	ΔE_vdW_	ΔE_elec_	ΔG_GB_	ΔG_SA_	ΔG_bind_
IFI27/Acetohexamide	-12.74 ± 1.21	-22.66 ± 1.37	16.99 ± 2.03	-1.51 ± 0.23	-19.91 ± 0.98
IFI27/Resiniferatoxin	-40.23 ± 2.24	-2.29 ± 1.63	15.33 ± 1.59	-5.80 ± 0.24	-32.98 ± 1.74

ΔE_vdW_, van der Waals energy; ΔE_elec_, electrostatic energy; ΔG_GB_, electrostatic contribution to solvation; ΔG_SA_, non-polar contribution to solvation; ΔG_bind_, binding free energy.

Hydrogen bonds are one of the strongest non-covalent interactions, and a higher number of hydrogen bonds indicates better binding. As shown in **Figure 7F**, the IFI27/Acetohexamide complex had fewer hydrogen bonds (around 1) during the simulation, indicating a weak contribution of hydrogen bonds to their binding. In contrast, the IFI27/Resiniferatoxin complex had around 2 hydrogen bonds during the simulation, suggesting that hydrogen bonds play a significant role in their binding.

### 3.12 Drug administration experiment in mice

**Figure 8:**
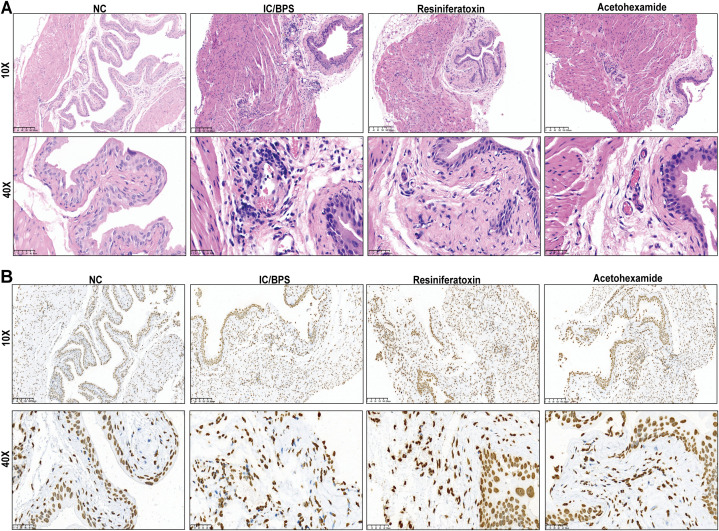
Validation of mouse administration experiments for two drug candidates. **(A)** Representative H&E staining in different subgroups. **(B)** Representative immunohistochemical staining images of IFI27 expressed in different subgroups.

**Figure 9:**
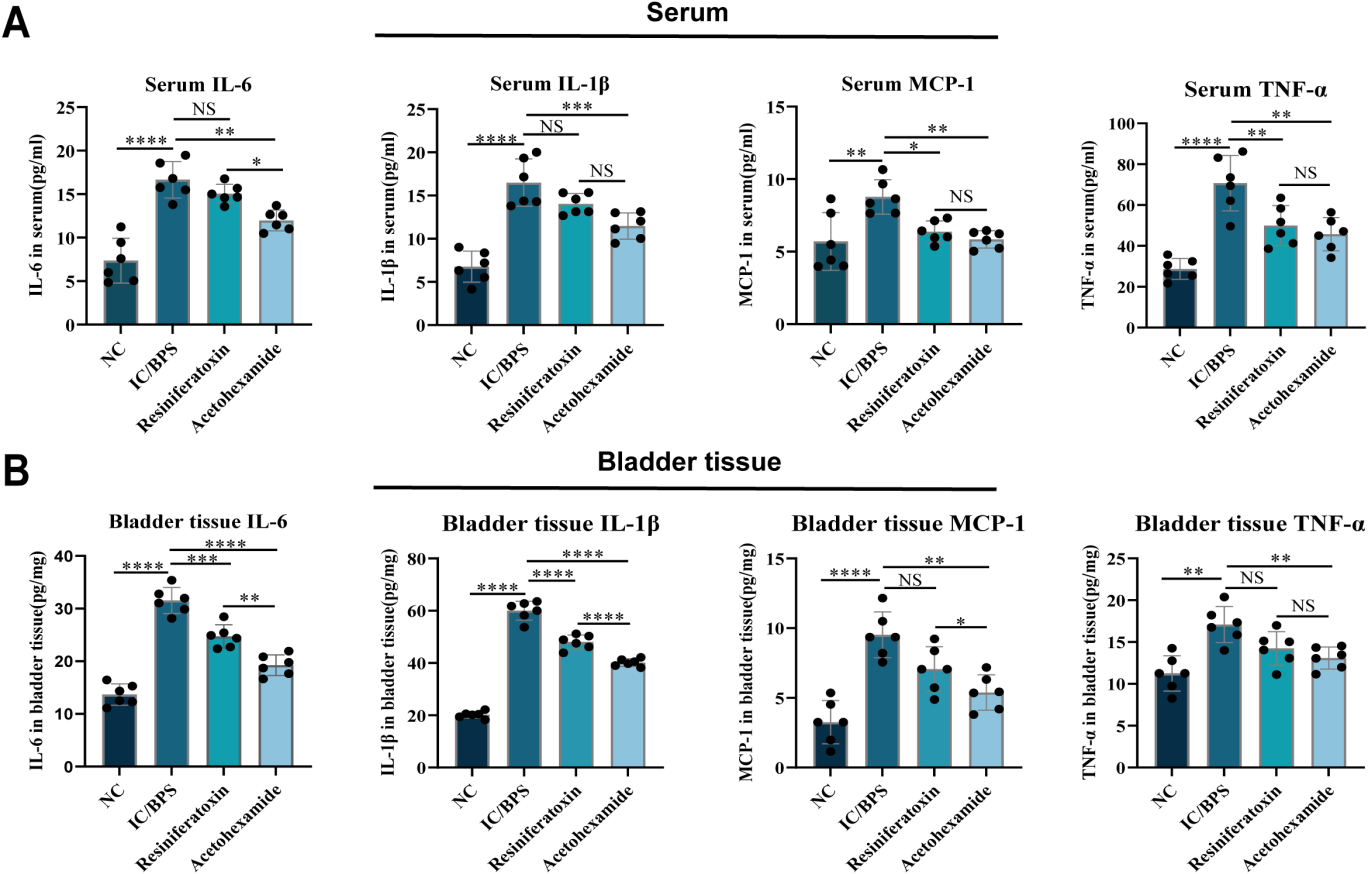
ELISA to verify changes in inflammatory factor levels in serum and bladder tissue before and after administration. **(A)** Serum; **(B)** bladder tissue. *p < 0.05; **p < 0.01; ***p < 0.001; ****p < 0.0001; NS, no statistical difference.

To validate the therapeutic efficacy of IFI27 and candidate drugs, histopathological evaluation of IC/BPS models revealed significant inflammatory cell infiltration within the lamina propria, accompanied by mild mucosal edema and partial epithelial denudation characterized by intercellular space widening (**Figure 8A**). Resiniferatoxin treatment marginally attenuated inflammatory infiltration, whereas acetohexamide markedly reduced mucosal edema and inflammatory infiltration. Mechanistically, upregulated IFI27 expression in IC/BPS models confirmed the pivotal role of innate immune activation, with immunohistochemical analysis demonstrating acetohexamide’s superior efficacy in downregulating IFI27 compared to resiniferatoxin (**Figure 8B**). Given the characteristic immune dysregulation in IC/BPS, cytokine profiling via ELISA revealed significantly elevated IL-6 and TNF-α levels in serum and bladder tissues of model groups. Both therapeutics exhibited anti-inflammatory effects, yet acetohexamide demonstrated significantly stronger suppression of proinflammatory cytokines than resiniferatoxin (**Figures 9A, B**).

## 4 Discussion

IC/BPS is a refractory disease with a complex etiology, characterized by severe pelvic pain and urinary symptoms. Due to the complexity of its disease process, IC/BPS lacks reliable characteristic biomarkers or effective treatments. Previous studies have demonstrated that abnormal immune responses are a significant histological feature of IC/BPS, with both innate and adaptive immune mechanisms potentially influencing its pathogenesis and progression. Therefore, exploring immune cell infiltration and immune-related genes is of great importance for understanding IC/BPS.

In this study, we integrated publicly available datasets from the GEO database, combining gene expression profiles from 31 IC/BPS samples and 14 normal controls. WGCNA was employed to filter out 27 Immune-related IC/BPS-DEGs. Based on 113 different machine learning algorithms, 8 immune-related IC/BPS signature genes were identified for further investigation. Additionally, GSVA revealed associations of these genes with pathways such as cytokine-cytokine receptor interaction, chemokine signaling, and NOD-like receptor signaling, indicating abnormal connections with immune responses and immune cell recruitment.

Existing evidence suggests that these eight core genes exhibit multifaceted functions in immune regulatory networks, and their aberrant expression may contribute to the pathological processes of IC/BPS through various immune mechanisms. IFI27, a member of the interferon-induced gene family, plays a central role in antiviral innate immunity by activating the RIG-I signaling pathway (15). Studies have shown that it not only inhibits dengue virus replication but also influences immune infiltration characteristics of monocytes and plasmacytoid dendritic cells by regulating the JAK-STAT pathway, highlighting its dual role in immune microenvironment remodeling (16, 17). CSF2RB, the common beta chain of the IL-3/IL-5/GM-CSF receptor, participates in inflammatory responses by regulating monocyte-macrophage differentiation (18). Overexpression of CSF2RB in inflammatory bowel disease may exacerbate immune homeostasis imbalance by altering gut microbiota balance. CDC25B, a key cell cycle regulator, promotes tumor cell proliferation through the MAPK pathway, while its expression levels positively correlate with M1 macrophage and activated dendritic cell infiltration in the tumor microenvironment, suggesting its potential role in coordinating cell cycle progression and immune responses in anti-tumor immunity (19, 20). DUSP5, a negative regulator of MAPK signaling, may modulate T cell activation thresholds by dephosphorylating ERK, although its specific role in the immune microenvironment requires further validation (21). IGSF3 in glioma inhibits Kir4.1-mediated potassium clearance, leading to neuronal depolarization and epileptiform discharges. This ion homeostasis imbalance may exacerbate neuroinflammation by activating microglia (22, 23). WNK3 regulates cell volume through ion cotransporters such as NKCC1, and its interaction with NKCC1 may be involved in intracellular chloride concentration regulation during T cell activation, suggesting its potential role in immune cell migration and effector functions (24, 25). Additionally, studies have shown that DLG2 expression is downregulated under inflammatory conditions, affecting inflammasome activation (26). Although DCDC2 is primarily associated with neuronal development, recent research has linked its polymorphisms to susceptibility to autoimmune diseases, potentially influencing immune system function through neuro-immune axis crosstalk (27). These genes form a regulatory network involving multiple levels, including innate immune signaling activation, cytokine responses, cell cycle regulation, and ion homeostasis maintenance, all of which are closely related to the dynamic balance of the immune microenvironment.

In this study, immune infiltration analysis based on the CIBERSORT algorithm revealed that the expression patterns of IC/BPS core genes were significantly correlated with the activation states of various immune cell subsets, particularly showing positive correlations with T cells CD4 memory activated, T follicular helper (Tfh) cells, regulatory T cells (Tregs), and M0/M1 macrophages, while negatively correlating with anti-inflammatory M2 macrophages. This finding suggests a significant pro-inflammatory bias and immune regulatory imbalance in the IC/BPS immune microenvironment. Previous studies have indicated that CD4+ T cell subsets in IC/BPS patients are highly heterogeneous, with Th1 cells activated by antigen-presenting cells through T cell receptor signaling, while Tregs exhibit a tendency to differentiate toward Th1 (28). This Treg dysfunction may weaken immune suppression, exacerbating local inflammatory responses. Additionally, significant Tfh cell infiltration is closely related to the enrichment of autoimmune disease pathways, potentially promoting B cell activation and antibody production in the formation of Hunner-type lesions (29, 30). Notably, macrophage polarization plays a critical role in disease progression, with the enrichment of M0/M1 macrophages associated with the release of pro-inflammatory factors (e.g., IL-1β, TNF-α) and neutrophil recruitment, while the reduction of M2 macrophages may impair tissue repair, collectively contributing to persistent inflammation and fibrosis of the bladder mucosa (31). This phenomenon is consistent with serological evidence—elevated levels of inflammatory markers such as CRP and IL-6 in IC/BPS patients suggest a synergistic effect between systemic inflammation and the local immune microenvironment. Overall, the pathological mechanisms of IC/BPS involve T cell subset dysfunction, macrophage polarization imbalance, and multi-dimensional inflammatory mediator network activation, collectively driving chronic inflammation and clinical heterogeneity.

Based on gene enrichment in transcriptomic data and qRT-PCR results, we selected IFI27 as a drug target and identified two small-molecule compounds with strong and stable binding affinity using AutoDock Vina. Acetohexamide, also known as pentosan polysulfate sodium, is a synthetic heparinoid drug (32). A core pathology of IC/BPS is the defect in the glycosaminoglycan (GAG) layer of the bladder mucosa, allowing irritants (e.g., potassium ions) in urine to penetrate the bladder wall, triggering pain and inflammation (33). The core mechanism of acetohexamide involves its GAG-mimicking structure, which directly binds to and repairs defects within the GAG layer of the bladder urothelium, thereby reconstructing the critical urine-tissue barrier. This restoration of the barrier fundamentally prevents the penetration of urinary irritants into deeper bladder wall layers, reducing their persistent stimulation of sensory nerve endings and immune cells. Furthermore, acetohexamide’s inhibitory effect on mast cell activation significantly reduces the release of mediators such as histamine and tryptase (34). Our data suggests that treatment with acetohexamide reduced the level of inflammation in cystitis, and HE staining showed improved infiltration of inflammatory cells in bladder tissue. Clinical trials have shown that approximately 30-40% of patients experience significant improvement in pain and urinary frequency after 3-6 months of continuous treatment (35). Resiniferatoxin is an ultra-potent TRPV1 receptor agonist. TRPV1 receptors are highly expressed in C-fiber sensory nerves of the bladder and are closely associated with bladder pain transmission and inflammatory responses (36). Resiniferatoxin initially activates TRPV1 receptors, causing calcium influx and the release of neuropeptides such as substance P from nerve terminals, followed by receptor desensitization and functional inhibition of sensory nerve fibers. Long-term use can reduce bladder sensory nerve hypersensitivity (37). Resiniferatoxin has been used for intravesical instillation in treating overactive bladder, showing efficacy in relieving urgency and frequency (38). Therefore, it holds the potential for alleviating IC/BPS symptoms.

It is necessary to admit that there are still some limitations in this study. First, the sample size is relatively small, and additional IC/BPS datasets are needed to validate our findings in larger cohorts. Secondly, although 2 small molecule compounds with potential therapeutic effects on IC/BPS were predicted and their efficacy was preliminarily validated in animal models, the alleviating effects of Resiniferatoxin on pain symptoms remain to be confirmed in long-term clinical trials. Therefore, future studies should include experimental validation of more small molecule compounds as well as *in vitro* validation at the protein level. Additionally, the specific mechanisms of IRICs in IC/BPS should be further explored.

## 5 Conclusions

In this study, we systematically resolved the immune signature of IC/BPS by integrating transcriptomics, single-cell sequencing, and machine learning algorithms. The study innovatively combines WGCNA with integrated machine learning to overcome the limitation that traditional differential analysis is susceptible to background noise interference. The diagnostic models constructed from the eight core genes obtained from the screening had AUCs above 0.9 in the external validation set, which were significantly better than a single biomarker. Among them, IFI27, as a key regulator, was involved in the disease process by activating the RIG-I signaling pathway and modulating macrophage polarization. Drug prediction revealed that Acetohexamide and Resiniferatoxin have a high affinity for IFI27, and molecular dynamics simulations confirmed their binding stability, providing a structural basis for the new use of old drugs. Finally, *in vivo* experiments validated and compared the inflammatory inhibitory effects of the two drugs on IC/BPS. This study provides an important theoretical basis for the development of diagnostic and targeted therapies for IC/BPS with significant clinical translational potential.

## Data Availability

The datasets presented in this study can be found in online repositories. The names of the repository/repositories and accession number(s) can be found in the article/**Supplementary Material**.

## Supplementary material

Supplementary Figure 1 Dataset merge and calibration. **(A)** Box plot of the integrated dataset before calibration **(B)** and after calibration. **(C)** PCA map of the integrated dataset before calibration and **(D)** after calibration. PCA, principal component analysis.

Supplementary Figure 2 Variance analysis of integrated datasets. **(A)** Heat map of DEGs. **(B)** Schematic of a volcano.

Supplementary Figure 3 Enrichment analysis of immune-related differential genes after initial screening. **(A, B)** GO enrichment analysis. **(C)** KEGG enrichment analysis.
