# Supplementary material for: Integrated identification of immune-related therapeutic targets for interstitial cystitis via multi-algorithm machine learning: transcriptomic profiling and in vivo experimental validation
Source: Front Immunol. 2025 Jul 24;16:1636855. doi: 10.3389/fimmu.2025.1636855 (PMC12328188; doi:10.3389/fimmu.2025.1636855)
Supplement: Supplementary file 1 [file Table1.docx]

Supplementary Table 1. Sequences of qRT-PCR primers for core genes.

| **Gene** | **Forward primer sequence (5'-3')** | **Reverse primer sequence (5'-3')** |
| --- | --- | --- |
| IFI27 | GCCTCTGCTCTCACCTCATC | TGGCCACAACTCCTCCAATC |
| CDC25B | ACGCACCTATCCCTGTCTC | CTGGAAGCGTCTGATGGCAA |
| CSF2RB | GTCCAGGTGGGAGGGATTTG | CATCTAATCGGGGAGACGGC |
| IGSF3 | GCCGAGATTTCATGCTTCACT | CGGAACGCTTTCGGGTCATA |
| DUSP5 | GCGACCCACCTACACTACAAA | CTTCATAAGGTAAGCCATGCAGA |
| WNK3 | TGTTGAAATGACGGAAGATGACA | TCTGCCACTAGGAGAAGTAGC |
| DLG2 | CCTCTACGTCAGAGCCATGTT | ATCGGGCACGTTCCTTTCTTT |
| DCDC2 | CCAGCTTCTCGCCTCCTTATC | GGCCTTCTCATCGTTGACTTG |
