# Supplementary figures and images for: Integrated identification of immune-related therapeutic targets for interstitial cystitis via multi-algorithm machine learning: transcriptomic profiling and in vivo experimental validation
Source: Front Immunol. 2025 Jul 24;16:1636855. doi: 10.3389/fimmu.2025.1636855 (PMC12328188; doi:10.3389/fimmu.2025.1636855)

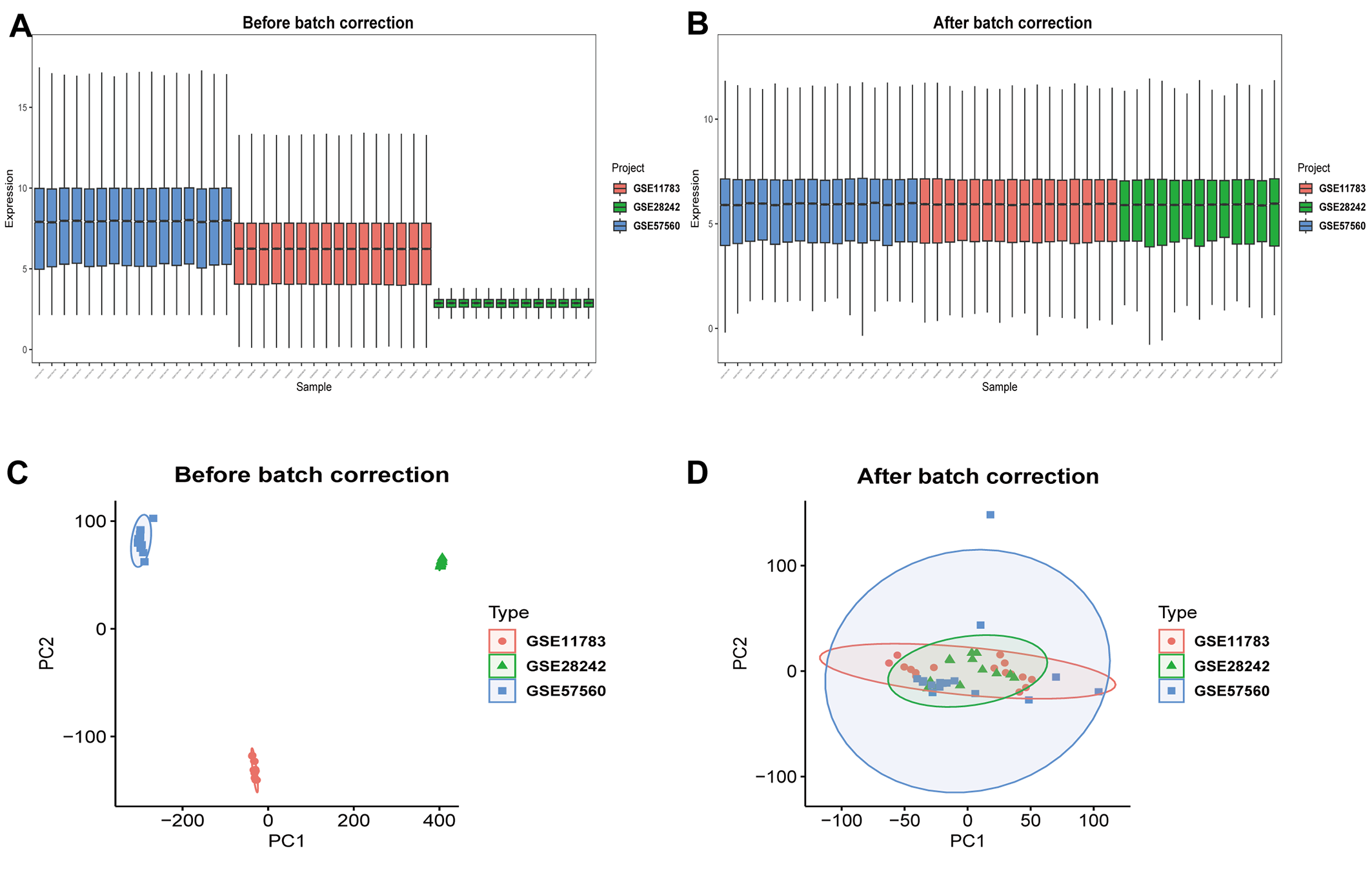

Supplement: Supplementary Figure 1 — Dataset merge and calibration. (A) Box plot of the integrated dataset before calibration (B) and after calibration. (C) PCA map of the integrated dataset before calibration and (D) after calibration. PCA, principal component analysis. [file Image1.tif]

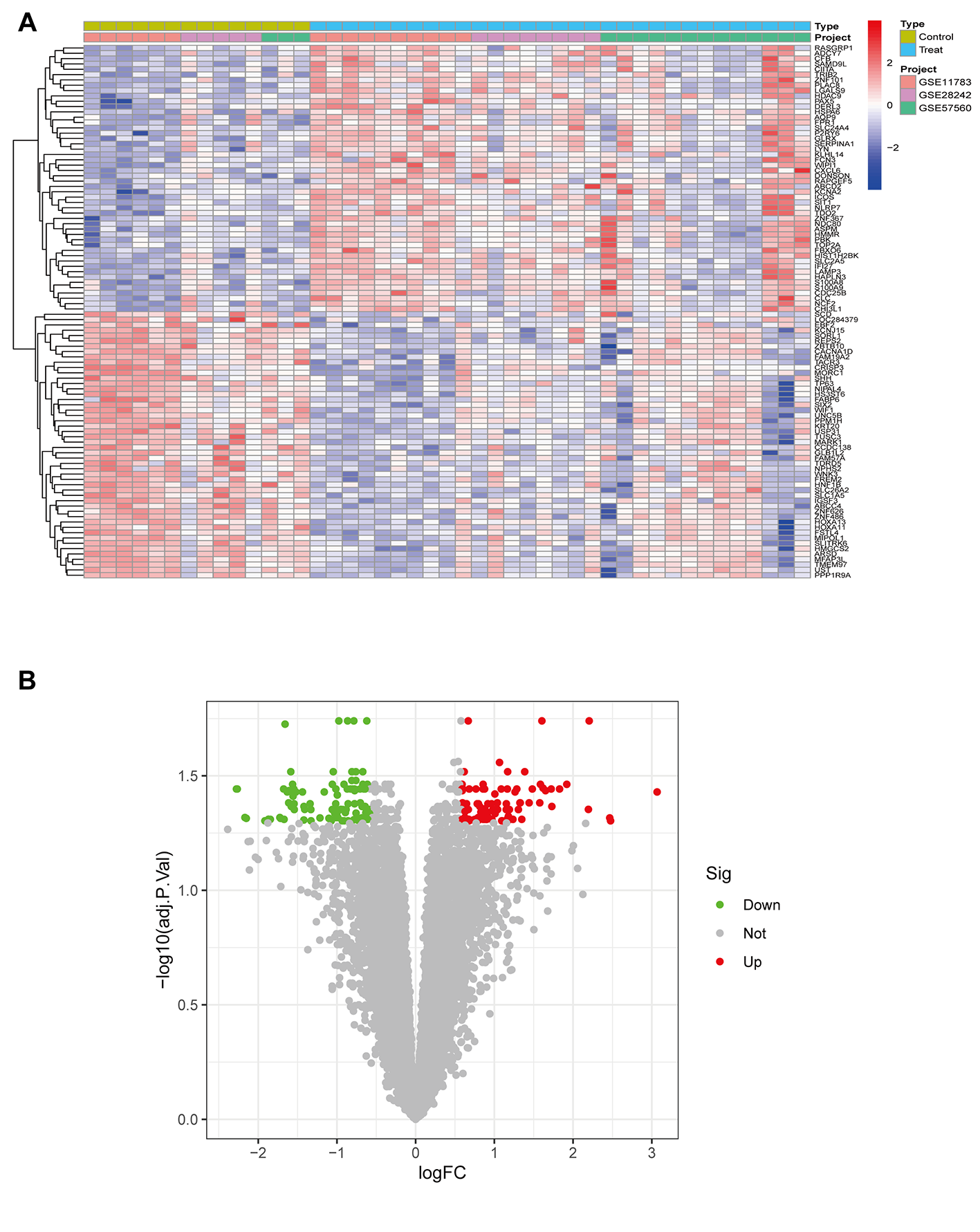

Supplement: Supplementary Figure 2 — Variance analysis of integrated datasets. (A) Heat map of DEGs. (B) Schematic of a volcano. [file Image2.tif]

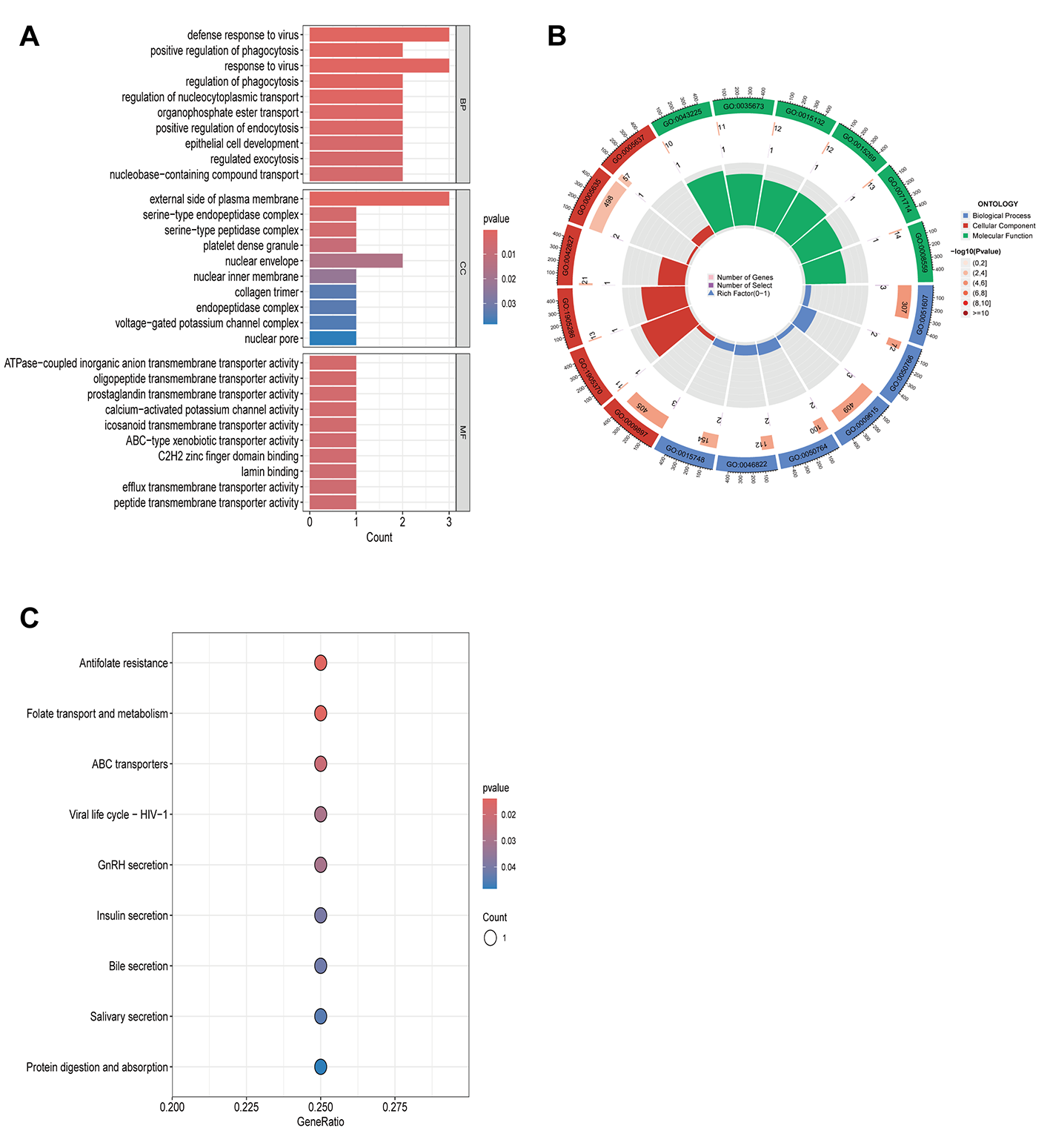

Supplement: Supplementary Figure 3 — Enrichment analysis of immune-related differential genes after initial screening. (A, B) GO enrichment analysis. (C) KEGG enrichment analysis. [file Image3.tif]
